# Impact of exosomes in oral lichen planus: A review with insights into pathogenesis and biomarkers

**DOI:** 10.1016/j.jds.2024.05.017

**Published:** 2024-05-18

**Authors:** Ram Mohan Ram Kumar, Suresh Joghee, Mahesh Kagarae Puttaraju

**Affiliations:** a Department of Pharmaceutical Biotechnology, JSS College of Pharmacy, JSS Academy of Higher Education & Research, Mysuru, Karnataka, India; b Department of Pharmacognosy, JSS College of Pharmacy, JSS Academy of Higher Education & Research, Mysuru, Karnataka, India; c Department of Oral Medicine and Radiology, JSS Dental College & Hospital, JSS Academy of Higher Education & Research, Mysuru, Karnataka, India

**Keywords:** Extracellular vesicles, Exosomes, Oral lichen planus, Biomarkers, Review

## Abstract

Oral Lichen Planus (OLP) presents a significant challenge in diagnosis due to its varied clinical manifestations and the absence of specific biomarkers. Timely and accurate diagnosis is crucial, particularly given its association with oral squamous cell carcinoma (OSCC). This review aims to explore the potential role of exosomes, small extracellular vesicles, in the pathogenesis of OLP and their utility as diagnostic biomarkers. Exosomes facilitate the exchange of information between cells and modulate immune responses by carrying various bioactive molecules such as proteins, lipids, and nucleic acids. In the context of OLP, exosomes derived from affected tissues or immune cells are thought to contribute to disease progression by mediating the transfer of pro-inflammatory molecules, including cytokines like interleukin-6 and tumour necrosis factor-alpha and chemokines such as CCL2, CCL5 and microRNAs such as miR-155, miR-146a, miR-21, and miR-34a, etc. Additionally, the distinct molecular contents of exosomes derived from OLP lesions may accurately represent the pathological changes occurring in these tissues. This suggests the potential of exosomes to be used as non-invasive biomarkers for diagnosing and tracking the progression of the disease. Understanding the immune microenvironment of OLP and the role of exosomes within this context is critical for advancing our knowledge of OLP pathogenesis and identifying new diagnostic and therapeutic strategies. However, challenges remain in identifying and characterising exosomes and their clinical translation. Further research is warranted to address these challenges and fully exploit exosomes' diagnostic and therapeutic potential in OLP and other inflammatory oral diseases.

## Introduction

Oral lichen planus (OLP) is a chronic inflammatory condition that affects the mucous membranes lining the oral cavity. Its clinical presentation is complex, with various lesions such as reticular, papular, plaque-like, atrophic, erosive, and ulcerative, each with distinct characteristics.^1^ Although the mechanisms leading to the formation of OLP have been extensively studied, the exact cause of the disease remains elusive. It is believed to arise from various factors, including genetic predisposition, immune system dysregulation, environmental influences, and potential triggers such as specific medications or systemic illnesses.^2^ Immunologically, OLP is highlighted by T-cell-mediated immune responses directed against antigens within the oral mucosa. This immune dysregulation and inflammation play an essential role in the initiation and progression of the disease.^3^ However, the antigens inciting this immune response in OLP have not been identified yet. The diagnostic process for OLP presents numerous challenges owing to its heterogeneous clinical manifestations and the absence of definitive diagnostic tests. While clinical examination remains fundamental, identifying hallmark features such as Wickham's striae in reticular lesions in OLP and differential diagnosis is essential.^4^ OLP lesions can mimic other oral mucosal conditions, including oral lichenoid reactions, autoimmune blistering disorders, and potentially precancerous or malignant lesions.^5^ Sometimes, a biopsy for histopathological examination may be necessary to confirm the diagnosis. However, this invasive procedure may not always be preferred, particularly for individuals with asymptomatic or low-risk lesions. Therefore, there is an unmet need for non-invasive, reliable biomarkers to facilitate accurate and timely diagnosis of OLP and predict treatment outcomes.

Exosomes are small extracellular vesicles (sEVs) secreted by cells. They typically range from 30 to 150 nm in diameter and encapsulate a diverse cargo of bioactive molecules, including proteins, lipids, nucleic acids, and metabolites. They are formed within the endosomal compartment of cells through a highly regulated biogenesis of exosomes.^6^ As scientific investigations delve deeper into the complex functions of exosomes in biological processes, it is increasingly apparent that their unique modifications play significant roles in the pathological processes associated with oral diseases. In the initial phases, the analysis of exosomal information aids in the early detection and recognition of oral diseases. As these diseases evolve and their prognosis becomes clearer, understanding the implications of exosome-related changes enhances our understanding of treatment selection and efficacy. Consequently, exosomes emerge as crucial indicators in diagnosing, monitoring, and managing various oral diseases, including OLP.

## Methods

A literature review based on PubMed publications related to OLP, exosomes, cancer, microRNA, biomarkers, pathogenesis, etc., was performed to summarize the role and impact of exosomes in OLP. The methods employed for this study involved a systematic search across four prominent databases: Scopus, Web of Science, PubMed, and Science Direct, as well as relevant clinical dataset websites. The search utilized a set of keywords including “oral lichen planus”, “exosomes”, “cancer”, “microRNA”, “biomarkers”, and “pathogenesis ", specifically targeting titles and abstracts. Additionally, relevant papers were sought from the reference lists of retrieved articles to ensure comprehensive coverage. In total, 158 articles were identified through this process, of which 32 were deemed pertinent to the study's objectives and included for further analysis.

### Exosome biogenesis, isolation and characterization

**Figure 1:**
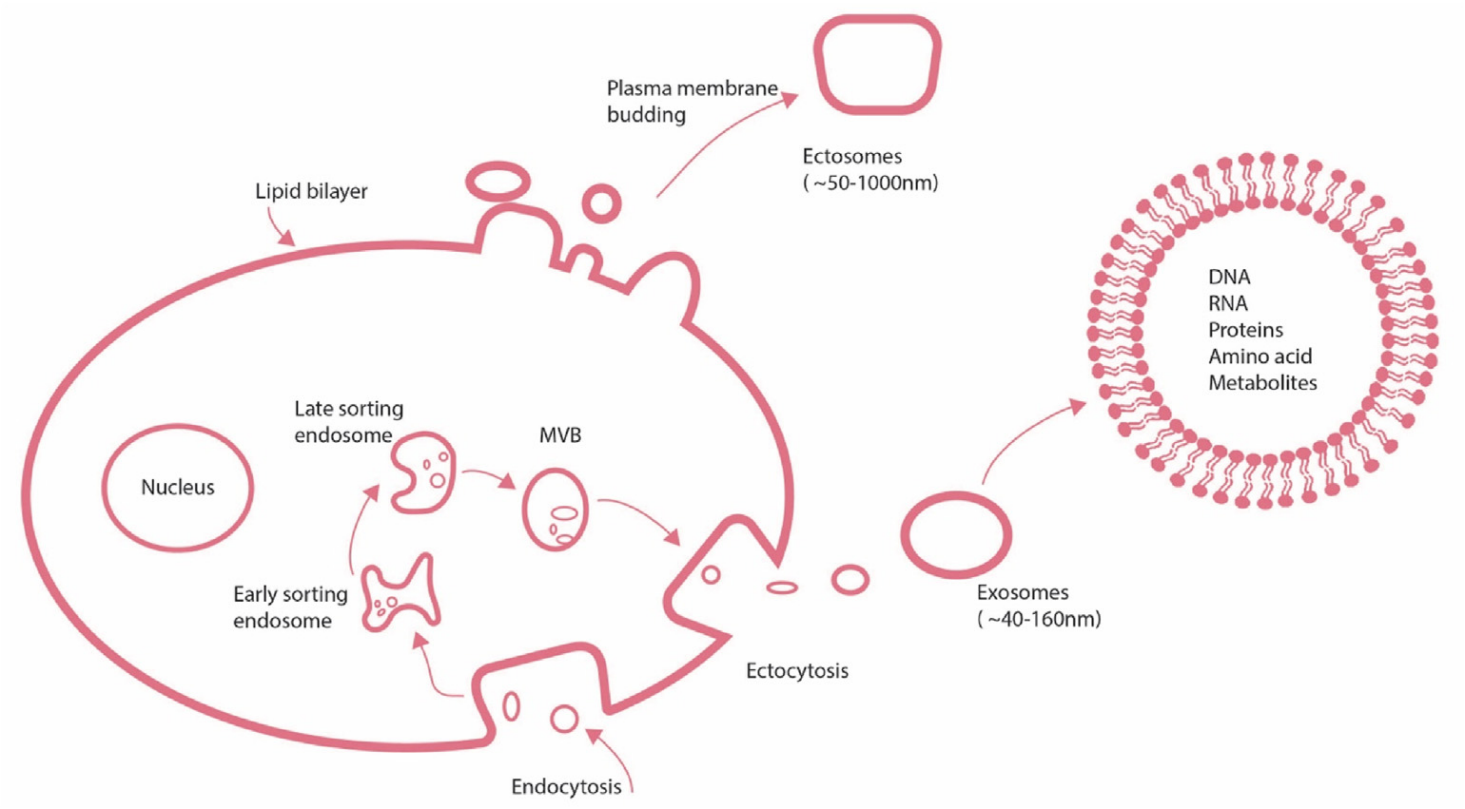
Mechanisms of exosome biogenesis and secretion. Exosome biogenesis begins with endocytosis, whereby the cell membrane undergoes inward budding, encapsulating bioactive molecules and forming endosomes. Within these endosomes, molecules are sorted into smaller vesicles, which bud from the perimeter membrane into the endosome lumen, forming multivesicular bodies (MVBs). Subsequently, MVBs can either fuse with lysosomes for degradation or fuse with the plasma membrane, releasing exosomes. The process of exosome formation from MVBs occurs through ESCRT-dependent and ESCRT-independent pathways.

Exosomes are small vesicles crucial for cellular communication, originating from the budding of early endosomes, formed through the inward budding of the cell membrane. As these endosomes mature, they transform into multivesicular bodies (MVBs) or late endosomes.^7^ This transformation involves a series of steps, including acidification of the endosomal lumen facilitated by proton pumps, which activates enzymes aiding cargo sorting and membrane restructuring.^8^ Sorting cargo molecules into intraluminal vesicles (ILVs) within MVBs is pivotal to exosome formation. This sorting process is mediated by the endosomal sorting complex required for transport (ESCRT) machinery, comprising several protein complexes and associated proteins. ESCRT-0 initiates cargo recognition and clustering by binding to ubiquitinated proteins on the endosomal membrane. ESCRT-I and ESCRT-II aid in cargo sorting and membrane invagination. At the same time, ESCRT-III orchestrates ILV scission from the endosomal membrane, forming MVBs loaded with ILVs containing specific cargo molecules.^9^ Fig. 1 shows exosome biogenesis and secretion.

Apart from the ESCRT-dependent pathway, various ESCRT-independent mechanisms contribute to exosome formation, involving diverse proteins, lipids, and cellular processes. Tetraspanin proteins like CD9, CD63, and CD81 organize membrane microdomains, facilitating the sorting of specific cargo molecules into ILVs.^10^ Lipids, such as ceramide and cholesterol, are essential for shaping the membrane and initiating vesicle formation during exosome development. Various factors influence exosome formation and cargo sorting, including cellular stress, signalling pathways, and environmental signals. Conditions like hypoxia, oxidative stress, and inflammatory cytokines can alter exosomes' production and release.^11^ Signalling pathways like mitogen-activated protein kinase (MAPK) and PI3K/Akt are vital in controlling critical stages of exosome formation, such as selecting which cargo molecules to include and forming ILVs.^12^ Additionally, the composition of the extracellular environment, including factors like pH, calcium levels, and components of the extracellular matrix, can affect both the secretion of exosomes and the composition of their cargo.^13^

Various techniques are employed to characterize isolated exosomes, essential for understanding their biological functions and potential clinical applications. These techniques include nanoparticle tracking analysis (NTA) or dynamic light scattering (DLS) for size determination, transmission electron microscopy (TEM) for morphological assessment, and western blotting for profiling exosome proteins.^14^ High-resolution imaging techniques, like electron microscopy (EM) and atomic force microscopy (AFM), help understand exosome morphology. On the other hand, mass spectrometry-based proteomics approaches have emerged as powerful tools for dissecting the molecular contents of exosomes. Among these approaches, tandem mass tag (TMT) labelling in conjunction with liquid chromatography-mass spectrometry (LC-MS/MS) stands out for its ability to comprehensively profile the protein composition of exosomes. Using TMT labelling, researchers can simultaneously analyze multiple samples, enabling robust quantitative comparisons and identifying potential biomarkers or functional proteins within exosomes.^15^

### Pathogenesis and therapeutic options in oral lichen planus

The pathogenesis of OLP is a complex interplay of genetic predisposition, immune dysregulation, environmental and epithelial stress.^16^ Immune dysregulation with an aberrant T-cell-mediated immune response directed against antigens within the oral mucosa is another characteristic of OLP. Notably, CD8+ cytotoxic T lymphocytes (CTLs) play an essential role in OLP pathogenesis, destroying keratinocytes and contributing to the characteristic clinical features of the disease.^17^ The basal membrane degradation is initiated by chymases activating matrix metalloproteinase-9 (MMP-9), thereby facilitating the migration of CD8+ cytotoxic T lymphocytes into mucosal lesions.^18^ This characterises OLP as a T-lymphocyte-mediated chronic inflammatory mucosal disease. Another feature of OLP pathogenesis is immune dysregulation, characterized by aberrant activation of innate and adaptive immune responses. Inflammatory infiltrates, consisting primarily of T lymphocytes, are commonly found in the subepithelial layer of OLP lesions. These infiltrates release pro-inflammatory cytokines, such as interferon-gamma (IFN-γ), tumour necrosis factor-alpha (TNF-α), interleukin-6 (IL-6), and interleukin-17 (IL-17), which contribute to tissue damage and perpetuate the inflammatory cascade.^3^ Genetic factors have been implicated in OLP susceptibility, with human leukocyte antigen (HLA) alleles, particularly HLA-DR and HLA-DQ, showing associations with increased disease risk.^19^ Environmental triggers, such as viral infections (e.g., hepatitis C virus), medications (e.g., nonsteroidal anti-inflammatory drugs) and psychological stress, can cause OLP by eliciting or amplifying immune responses.^20^ Furthermore, epithelial stress or damage caused by microbial infections may trigger the initiation of OLP lesions by disrupting mucosal barrier integrity and facilitating immune cell infiltration.^21^ Despite significant progress in understanding OLP pathogenesis, many aspects are not entirely understood, underscoring the need for further research to unravel the intricate molecular mechanisms underlying this chronic inflammatory disorder. Such insights hold promise for developing targeted therapeutic strategies to modulate immune responses and restore mucosal homeostasis in OLP.

The involvement of extracellular vesicles (EVs) in the pathogenesis of OLP has been reported in a few studies. Elevated keratin 17 (KRT17) levels were observed in EVs originating from tissues affected by OLP, suggesting its potential involvement in pathogenesis. KRT17 is classified among human type I epithelial keratins, playing a role in maintaining epidermal integrity. It is predominantly expressed in basal cells of complex epithelial structures, like hair follicles. Consequently, KRT17 is a significant contributing factor in the pathogenesis of immune-related diseases.^22^ Sun et al. discovered the tissue-derived EVs (TDEVs) immunogen associated with developing OLP and its variation. The TDEVs were isolated from a pool of OLP patients, and proteomic analysis was performed. Of the ten differentially expressed proteins, protein disulfide isomerase family A member 3 (PDIA3) was upregulated in antigen processing and presentation pathway.^23^ This might play a significant role in the local immune responses and the pathogenesis of OLP.

Treatment options for OLP aim to alleviate symptoms, control inflammation, and promote mucosal healing. Topical corticosteroids provide potent anti-inflammatory effects by suppressing immune responses and reducing cytokine production. Intralesional corticosteroid injections are administered directly into lesions to achieve localized anti-inflammatory effects in cases of more severe or refractory OLP, reducing inflammation, alleviating pain, and promoting healing within the lesions.^24^ Systemic corticosteroids may be considered for widespread use; however, potential adverse effects limit their long-term use. Calcineurin inhibitors inhibit T-cell activation and cytokine production to mitigate inflammation. These agents are typically applied topically and are particularly useful in cases where corticosteroid therapy is ineffective.^25^ Another method involves the use of photodynamic therapy (PDT) as an adjunctive treatment for OLP. PDT consists of administering a photosensitizing agent followed by exposure to light, generating reactive oxygen species and selective destruction of targeted cells.^26^ Table 1 lists the current drugs, effect of action and side effects in treating OLP.

**Table 1:** Different drugs in treating OLP and its side effects.

Category	Drug	Effect of action	Side effects	References
Corticosteroids	Clobetasol propionate	Anti-inflammatory and immunosuppressive to OLP. Prevent erythema and ulceration.	Skin thinning	^27^
	Triamcinolone acetonide	Prevent erythema and ulceration.	Inflammation of hair follicles	^28^
	Fluocinonide	Prevents itching, burning, and discomfort.	Loss of skin pigmentation	^29^
	Betamethasone	Anti-inflammatory and immunosuppressive to OLP.	Dilation of small blood vessels near the surface of the skin	^30^
Immunomodulators	Tacrolimus	Inhibit T-cell activation.	Gastrointestinal disturbances	^31^
	Pimecrolimus	Reduces cytokine production and inflammation	Inflammation of the skin at the application site.	^32^
	Thalidomide	Inhibit the production of TNF-α, IL-6 and IFN-γ.	Nerve damage, numbness	^33^
	Tofacitinib	Reduces the activation and proliferation of T lymphocytes	Gastrointestinal disorders	^34^
	Methotrexate	Suppresses T lymphocyte activity	Gastrointestinal disorders	^35^

Abbreviations: OLP: oral lichen planus.

### Role of exosomes in immune regulation in oral lichen planus

**Figure 2:**
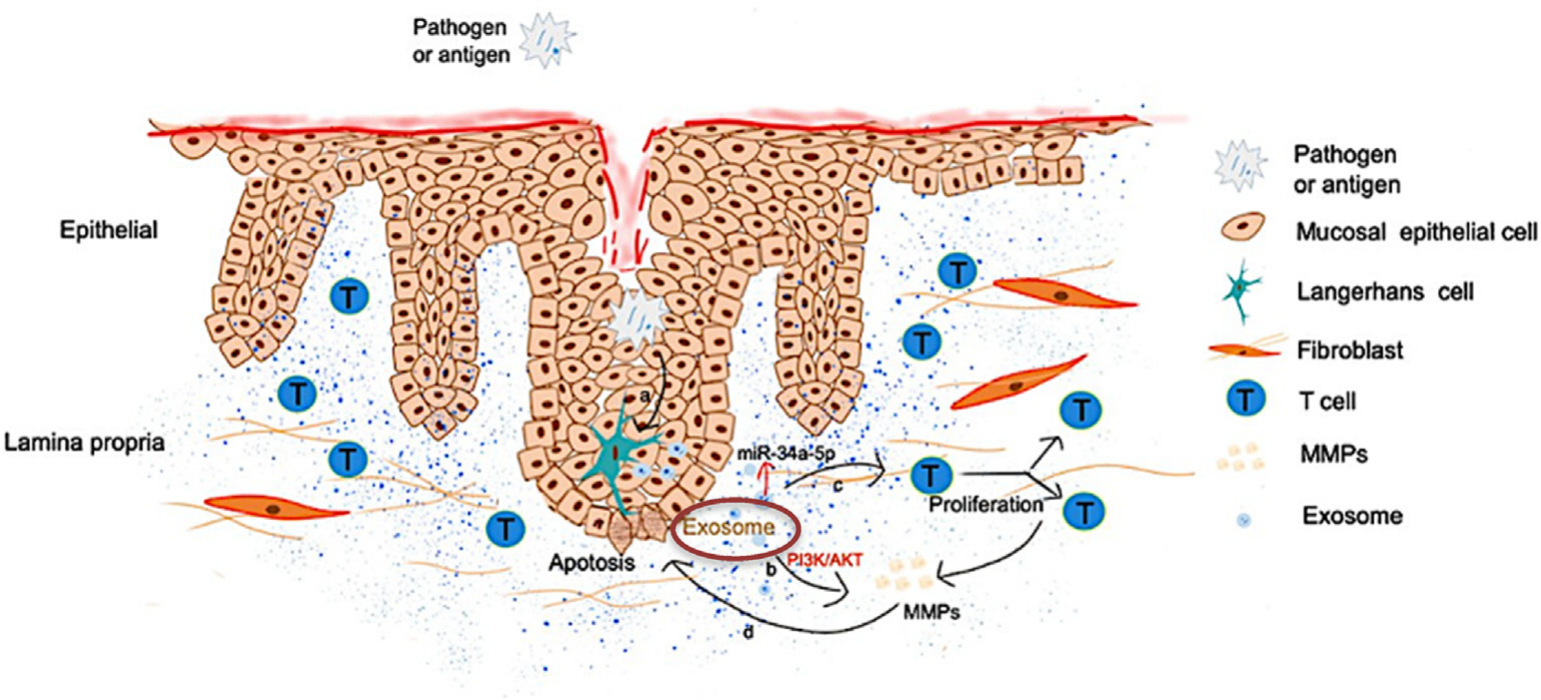
Immune regulation by exosomes in OLP: When pathogens or antigens penetrate the mucosal epithelium, they trigger Langerhans cells to release exosomes. These exosomes carry miRNAs that infiltrate the lamina propria and induce the secretion of matrix metalloproteinases (MMPs) via the PI3K/AKT signaling pathways. The exosomal miRNAs further stimulate the proliferation of CD4+ and CD8+ T cells and the secretion of MMPs. MMPs, in turn, inhibit the proliferation of epithelial cells while promoting the apoptosis of keratinocytes.

Exosomes have been recently implicated in immune regulation and inflammation in OLP, influencing their impact on disease pathogenesis and progression (Fig. 2). Exosomes derived from immune cells, such as dendritic cells and T lymphocytes, have been identified as crucial mediators of immune activation and inflammation in OLP.^36^ Exosome-mediated transfer of inflammatory microRNAs has also been implicated in the activation of immune cells and the persistence of chronic inflammation in OLP lesions.^37^ Moreover, exosomes derived from OLP lesions exhibit unique molecular signatures, containing specific microRNAs and pro-inflammatory molecules that reflect the disease state and contribute to its pathogenesis. Studies have identified specific exosomal microRNAs derived from OLP lesions, such as miR-155, miR-146a, miR-21, and miR-34a, suggesting their involvement in OLP pathogenesis. These microRNAs target genes involved in inflammatory pathways such as Wnt/β-catenin, PI3K/AKT and NF-kB pathways, thereby contributing to the inflammation in OLP.^38^

Additionally, exosomes carry cytokines and chemokines that can modulate the activity of immune cells and promote an inflammatory response in OLP. Cytokines like interleukin-6 (IL-6), tumour necrosis factor-alpha (TNF-α), and chemokines such as CCL2 have been identified in exosomes isolated from OLP lesions.^39^ These molecules can stimulate immune cell activation and recruitment to the site of inflammation, further exacerbating tissue damage and inflammation in OLP. Yang et al. studied the effect of T cell-derived exosomes in the OLP pathogenesis. When T cell-derived exosomes were treated in Jurkat cells, high levels of expression of macrophage inflammatory protein (MIP)-1α/β were detected. Incidentally (MIP)-1α/β was upregulated in OLP tissues and plasma. In their study, MIP-1α/β influenced the migration of OLP mononuclear cells. Thus, T-cell-derived exosomes may induce the production of MIP-1α/β, which could facilitate the migration of CD8+ T cells upon interaction with CCR1/5 receptors, therefore playing a significant role in the pathogenesis of OLP.^36^ Peng et al. investigated the role of exosomes in T-cell regulation, where exosomes significantly enhanced T-cell proliferation and attenuated the apoptosis of T cells.^40^ Table 2 lists the exosome contents and their role in immune regulation in OLP.

**Table 2:** Exosome contents involved in immune regulation in OLP.

Exosomal Contents	Types	Effects in OLP	References
Cytokines	IL-6, TNF-α, IFN-γ	Induce inflammation and tissue damage in OLP lesions	^41^
Chemokines	MIP-1α/β, CCL5, CXCL10	Recruit immune cells to the site of inflammation	^36^
Major Histocompatibility Complex (MHC) molecules	MHC class I and II molecules, CD86, CD80	Facilitate antigen presentation and T-cell activation	^42^
Growth factors	TGF-β, EGF, PDGF	Modulate cell proliferation and differentiation	^43^
Lipids	Phosphatidylserine, ceramide, sphingomyelin	Influence cellular signaling pathways	^44^
Proteins	TCR	Involvement in T cell activation and antigen recognition	^36^
	FasL	Potential induction of apoptosis in target cells	^45^
	CTLA-4	Potential modulation of T cell activation and immune tolerance	^46^

Abbreviations: IL6: interleukin 6, TNF-α: tumor necrosis factor alpha, IFN- γ: interferon gamma, MIP-1α/β: macrophage inflammatory protein, CCL5: chemokine (C–C motif) ligand 5, CXCL10: C-X-C motif chemokine ligand 10, TGF-β: tumor growth factor beta, EGF: epidermal growth factor, PDGF: platelet-derived growth factor, TCR: T cell receptor, FasL: Fas ligand, CTLA-4: cytotoxic T-lymphocyte-associated protein 4.

### Exosomes as biomarkers for diagnosis of oral lichen planus

The identification of reliable biomarkers for OLP diagnosis remains a clinical challenge. Recent research has suggested that exosomes may serve as promising biomarkers for OLP due to their unique cargo composition and stability in bodily fluids. Byun et al. examined the salivary exosomal miRNA expression profile among individuals with OLP. Their analysis reported differences in the expression levels of miR-4484, miR-1246, and miR-1290 between OLP patients and healthy subjects. Notably, they observed a significant increase in the expression of exosomal miR-4484 in the saliva of individuals diagnosed with OLP, which could be used as a potential biomarker for OLP.^47^ Peng et al. discovered that in OLP patients, there was a notable increase in plasma-derived exosomal miR-34a-5p and miR-130b-3p, while exosomal miR-301b-3p levels were reduced. Moreover, they observed a direct relationship between the expression levels of exosomal miR-34a-5p and the severity of OLP.^40^ Sepideh et al. reported a high level of expression of exosomal miR-21 and miR-31 in the saliva of OLP patients, which were associated with the onset and progression of OLP.^48^ Mehdipour et al. found that exosomal miR-125a expression was reduced in the saliva of individuals with OLP compared to healthy volunteers. miR-125a is a known tumor suppressor and is downregulated in patients with OSCC, implying the importance of this miRNA to be used as a biomarker for diagnosing OLP.^49^ Exosomal miR-137 is downregulated in the saliva and tissue of OLP patients compared to healthy individuals; hence, determining the expression of miR-137 could potentially aid in diagnosing OLP.^38^ Stasio et al. investigated the potential miR biomarkers in five patients with OLP. Their study observed elevated levels of exosomal miR-21, miR-125b, miR-203, and miR-15b, while miR-27b was downregulated in saliva samples.^50^ Table 3 lists the OLP-derived exosomal markers.

**Table 3:** OLP-derived exosome markers.

Exosome contents	Type	Number of OLP patients	Effect	References
miRNAs	miR-21, miR-125b, miR-203, miR-15b, miR-27b	20 OLP patients vs. 20 healthy controls	Increased/decreased expression; potential diagnostic markers	^51^
	miR- 146a, miR-155	60 OLP patients vs. 15 healthy volunteers	Decreased expression	^49^
Proteins	SPRR1B	23 OLP patients	Increased levels; potential role in immune response	^52^
	MMP-8 and CTX I	30 OLP patients vs. 30 healthy volunteers	Collagen degradation in the mucosal layer	^53^
	FAM3B	–	Decreased expression	^54^
Tetraspanins	CD63, CD9, CD81	40 OLP patients	Altered expression; potential markers of exosome biogenesis	^36^

Abbreviations: SPRR1B, small proline-rich protein 1B; MMP-8, matrix metalloproteinase-8; CTX, cerebrotendinous xanthomatosis; FAM3B, FAM3 metabolism regulating signaling molecule B.

## Conclusions

In the past few years, there has been significant progress in understanding the immune microenvironment of OLP. However, many aspects still require further investigation, specifically, understanding how immune elements contribute to the persistent inflammation characteristic of OLP. Within the immune microenvironment of OLP, it is becoming increasingly evident that exosome components play a pivotal role in its pathogenesis. With their potential as biomarkers and therapeutic agents, exosomes have become a focal point of research in diagnosing and treating OLP. Despite understanding the role of exosomes in OLP, various challenges remain. The accurate identification, analysis, and synthesis of exosomes from OLP patients still lacks precision and convenience. Clinical trials are scarce for the exosome-related diagnosis in OLP, and more studies are needed to characterise and ascertain the pathogenic functions of exosomes originating from OLP.

## Declaration of competing interest

The authors have no conflicts of interest relevant to this article.
